# Electronic Structures of Twisted Bilayer InSe/InSe and Heterobilayer Graphene/InSe

**DOI:** 10.1021/acsomega.1c01562

**Published:** 2021-05-11

**Authors:** Xiaojing Yao, Xiuyun Zhang

**Affiliations:** † Department of Physics, Hebei Normal University, Shijiazhuang 050024, China; ‡ College of Physics Science and Technology, Yangzhou University, Yangzhou 225002, China

## Abstract

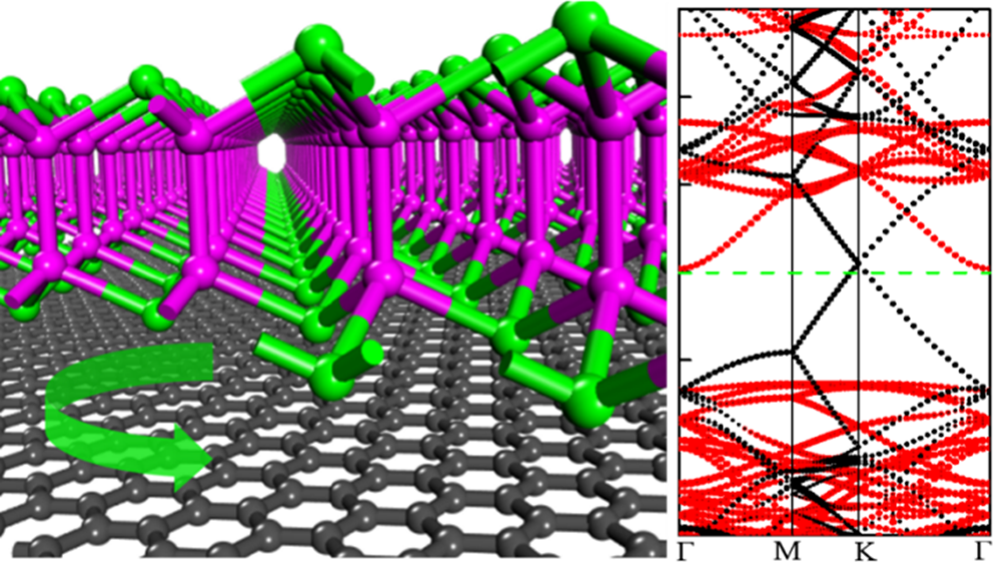

Building vertical van der Waals heterojunctions between two-dimensional layered materials has become a promising strategy for modulating the properties of two-dimensional materials. Herein, we investigate the electronic structures of non-twisted/twisted bilayer InSe/InSe and heterobilayer graphene/InSe (Gr/InSe) by employing density functional theory calculations. For twisted bilayer InSe/InSes, their interlayer distances and band gaps are almost identical but a bit larger than those of the AB-stacking one due to the spontaneous polarization. Differently, the band gaps of twisted Gr/InSe are found to vary with the rotation angles. Our results provide an effective way to tune the electronic properties of two-dimensional materials.

## 1 Introduction

To date, two-dimensional (2D) layered materials, such as graphene (Gr),^1^ silicene,^2^ transition metal dichalcogenides (TMDs),^3^ and black phosphorene (BP),^4^ have attracted great interest due to their excellent properties such as high carrier mobilities, strong quantum confinement effects, high on–off current ratios, etc., which enable them to be promising candidates in electronic and spintronic devices. Remarkably, the physical and chemical properties of 2D materials are manifested to be strongly dependent on their thickness. For example, linear band dispersion is found for monolayer graphene^5,6^ whereas it is changed to be quadratic in the bilayer system,^7^ making the band gap of the latter tunable by applying an external electric field.^8^ Similarly, the interlayer hopping in bilayer phosphorus creates a significant difference in the band gap with the monolayer.^9^ Corresponding to monolayer MoS_2_, a direct semiconductor, it is changed to be an indirect semiconductor for its bilayer isomer.^10^ In addition to band gap manipulation, it is revealed that the magnetic properties can be tuned by interlayer coupling. For example, in-plane magnetic orders of mono- and few-layer CrS_2_ can be switched between striped antiferromagnetic and ferromagnetic orders upon manipulating charge transfer between Cr t_2g_ and e_g_ orbitals.^11^ Interestingly, a tremendous amount of work has demonstrated that the electronic properties of bilayer 2D materials are largely dependent on their twist angles.^12−14^ For example, a flat band exhibiting insulating states at half-filling^15−17^ was revealed to appear near the Fermi level for the twisted bilayer graphene with “magic” angles. Moreover, the band gaps of twisted bilayer MoS_2_ can be tuned up to 5% when the twist angle varies from 21.8 to 0°.^18^ As for bilayer BP, it is found that the anisotropy of its electronic structure and optical transitions can be tuned by gating with an interlayer twist angle of 90°.^19^

Another effective way to expand the applications of 2D materials is to construct van der Waals (vdW) heterostructures by coupling hetero 2D materials. For example, the band gap of graphene can be opened by coupling it with other 2D monolayer systems like TMS_2_ (TM = Mo and W) heterobilayers,^20^*h*-BN,^21^ borophene,^22^ and so on, which makes it a potential candidate for electronic devices. Theoretical and experimental explorations demonstrated that the heterostructures composed of MoX_2_ and WX_2_ (X = S, Se, or Te) have type-II band alignments.^23,24^ Theoretical studies indicated that twisted bilayers of Gr/MoS_2_ show significant differences in band structures from the non-twisted ones with the appearance of the crossover between direct and indirect band gap and gap variations.^25,26^ In addition, when coupling graphene with BP, on one hand, their respective properties are preserved in the composed heterostructure; on the other hand, the band structure of the two-sided monolayer material can be tuned by the application of an external electrical field perpendicular to the 2D plane.^27,28^

Analogue to graphene, the newly emerging 2D indium selenium monolayer,^29−31^ InSe, displays a hexagonal honey structure. Bulk InSe is a direct band gap semiconductor with a gap of 1.25 eV,^32^ while the band gap of monolayer InSe is enlarged due to the quantum confinement effect.^33^ The high electron mobility, quantum Hall effect, and anomalous optical response^29−36^ of monolayer InSe enable it to be a competitive choice in electronic applications. Moreover, heterostructures combining InSe with other 2D materials like graphene, MoS_2_, black phosphorus, *h*-BN, etc., have been successfully synthesized experimentally.^37−42^ However, efforts to explore the physical properties of layer-coupled InSe are limited. In this work, we investigate the structural and electronic properties of InSe/InSe and graphene/InSe (Gr/InSe) vdW heterostructures with different rotation angles by first-principles calculations. Our results show that, for twisted bilayer InSe/InSe, the interlayer distances and band gaps are almost insensitive to the twist angles but a bit larger than those of the AB-stacking one. Meanwhile, for Gr/InSe, their electronic structures are found to vary with different rotation angles.

## 2 Computational Methods

All the calculations were performed in a Vienna *ab initio* simulation package (VASP) under the framework of plane-wave pseudopotential density functional theory (DFT) methods.^43,44^ The energy cutoff for the plane-wave expansion was set to 400 eV. The ion–electron interactions and exchange–correlation interactions were described by the projector-augmented wave (PAW) method^45^ and the generalized gradient approximation (GGA) with the Perdew–Burke–Ernzerhof (PBE) functional,^46^ respectively. To consider the interlayer van der Waals (vdW) interactions, the DFT-D2 method^47^ was employed. All the atoms were allowed to be fully relaxed until the Hellmann–Feynman forces on each atom were smaller than 0.01 eV/Å. The Brillouin zone integration was sampled by a *k*-point mesh of ∼0.06 Å^–1^ within the Monkhorst–Pack scheme.^48^ The vacuum region was set larger than 10 Å to eliminate artificial interactions in adjacent unit cells. Spin polarization was taken into account in all the calculations.

The configurations of twisted bilayer InSe/InSe and heterobilayer Gr/InSe were constructed according to accidental angular commensurations.^49^ For a hexagonal lattice with lattice vectors of ***a*_1_** and ***a*_2_**, its correspondent deflective supercell has a basis vector of *n**a*****_1_** + *m**a*****_2_** and a rotation angle (θ), as defined in Figure S1 in the Supporting Information
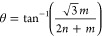
Therefore, the relative rotation angles (θ_r_) for the twisted InSe/InSe and Gr/InSe bilayer are defined as θ_r_ = θ_InSe-up_ – θ_InSe-down_ and θ_r_ = θ_Gr_ – θ_InSe_, respectively. We denoted the twisted heterobilayer Gr/InSe with a notation of *p*:*q*, where *p* and *q* are the periodicities of graphene and InSe layers, respectively.

The lattice constants of graphene and InSe are 2.47 and 4.09 Å, respectively, which are consistent with a previous study.^50^ In our calculations, the lattice constants of Gr/InSe supercells took the average values of the graphene and InSe monolayer, with the compressive and tensile strains on graphene or InSe are in the same magnitude and are smaller than 1%. We adopted three types of twisted Gr/InSe heterobilayers in this study, namely, 6:√13, √19:√7, and √43:4.

## 3 Results and Discussion

### 3.1 Bilayer InSe/InSe

**Figure 1:**
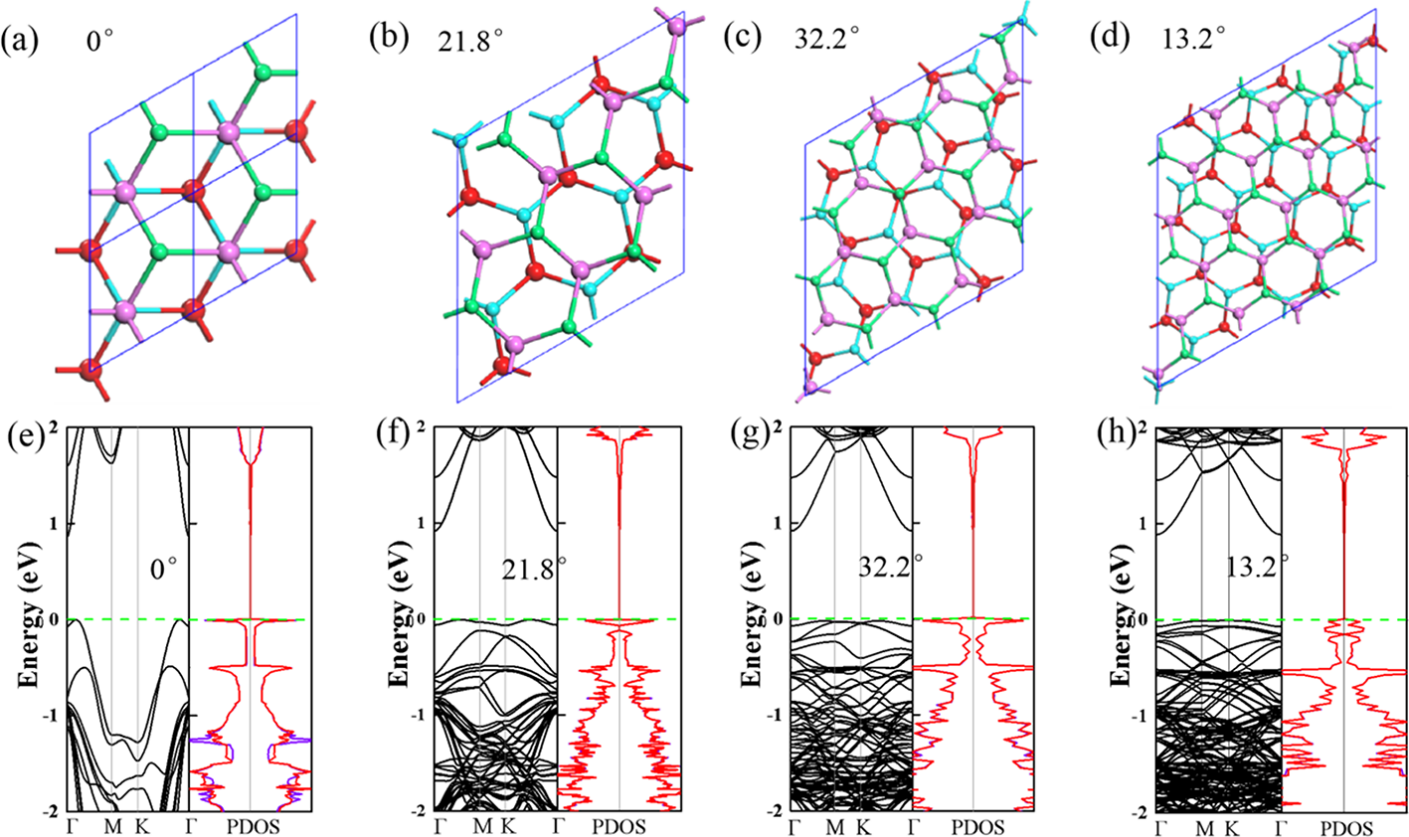
Optimized structures of (a) AB-stacking and (b–d) twisted InSe/InSe structures with interlayer rotation angles of 21.8, 32.2, and 13.2°, respectively. Magenta and green balls represent In and Se atoms in the upper layer, and red and cyan balls represent In and Se atoms in the bottom layer, respectively. (e–h) Band structure and PDOS for each type of bilayer InSe/InSes. Violet and red colors in PDOS denote the contributions from the top and bottom InSe layers, respectively.

Four types of bilayer InSe/InSe with θ_r_ = 0, 21.8, 32.2, and 13.2° (with supercells of 1, √7, √13, and √19, respectively) are considered (see Figure 1). The system with θ_r_ = 0° refers to Bernal stacking (AB-stacking) configuration without rotation between two InSe layers, which is much stable than the AA-stacking one (Figure S2 in the Supporting Information) by about 15.36 meV lower in the binding energy (*E*_b_), according to the following equation (eq 1)
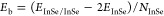
1where *E*_InSe/InSe_, *E*_InSe_, and *N*_InSe_ are the energies of bilayer InSe/InSe, monolayer InSe, and the number of atoms per unit cell, respectively. Since the stacking InSe/InSe with the same rotation angles may have different geometries due to the interlayer translation, here, we tested three interlayer registries for InSe/InSe with θ_r_ = 21.8°; that is, the Se atom from the top layer superimposes on a Se (Se–Se)/In atom (Se–In) or on the hollow site (Se–H) in the bottom layer (see Figure S3 in the Supporting Information). Our results indicate that the energies and band structures are almost insensitive to the configurations with a maximum energy difference of only 3 meV in the supercell, wherein the Se–In configuration has a relatively lower energy; therefore, we use the Se–In configuration model to explore the properties of twisted bilayer InSe/InSes below.

**Figure 2:**
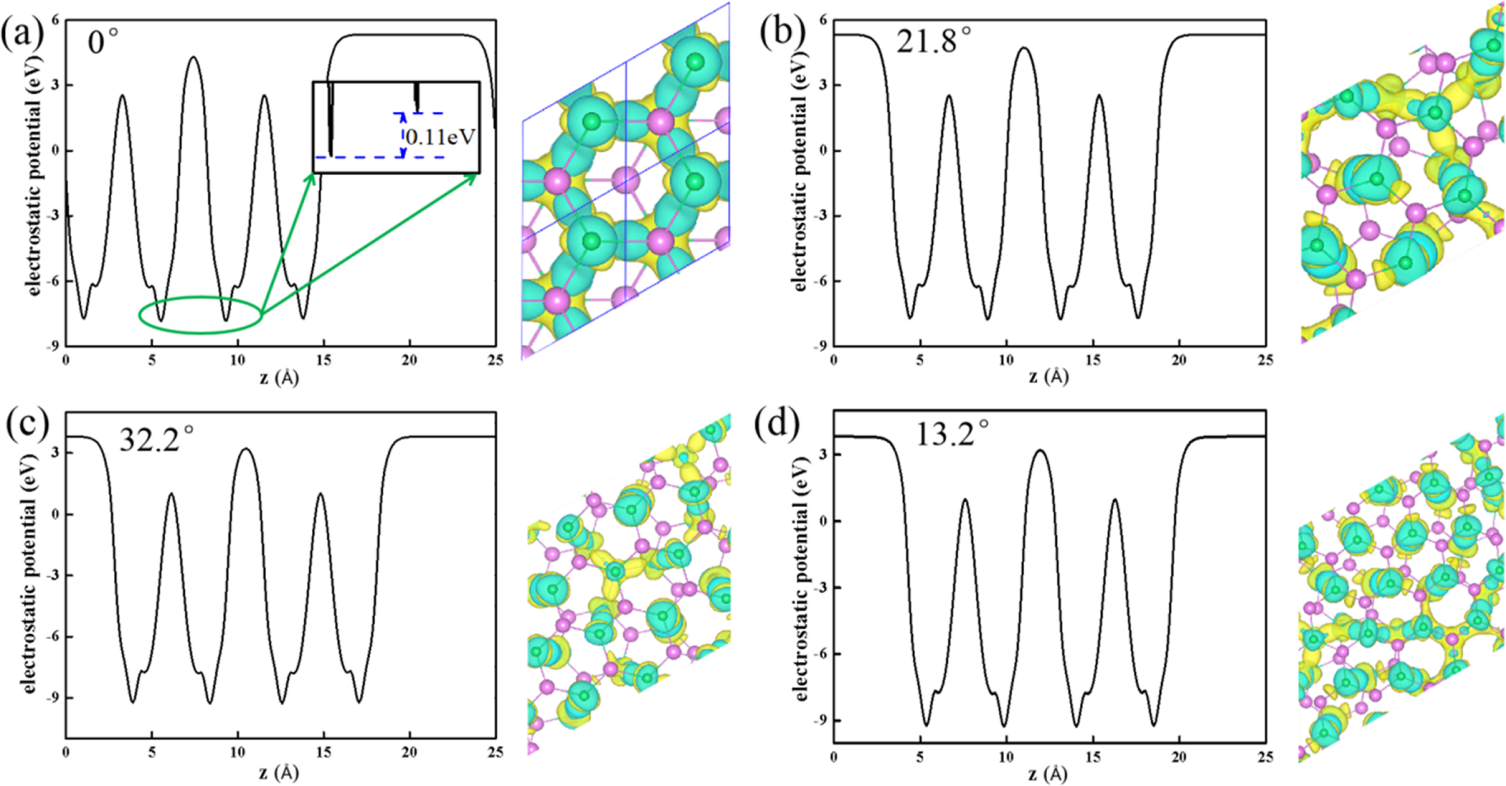
Electrostatic potential along the *z* direction and CDD plots for twisted InSe/InSe at θ_r_ = 0° (a), 21.8° (b), 32.2° (c), and 13.2° (d). Yellow and blue regions denote electron accumulation and depletion, respectively. The isosurface value is 1.2 × 10^–4^ e/bohr.^3^

The interlayer distances and the binding energies of the four studied systems are listed in Table 1. Compared with AB-stacking configuration (θ_r_ = 0°), the twisted bilayer InSe/InSe is less energetically favorable with the binding energies reduced by about 26–29%; as a result, the interlayer distances are increased by about 13–17%. Differently, no significant differences are found for the binding energies and the interlayer distances for the twisted InSe/InSes with various θ_r_ values. The charge density difference (CDD) of these bilayer InSe/InSes is calculated based on the following equation
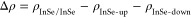
2The top and side views of the CDDs are shown in Figure 2 and Figure S4 in the Supporting Information. Clearly, the charges for all the bilayer InSe/InSes are accumulated between two InSe layers and depleted from the innermost Se atoms.

**Table 1:** Interlayer Twist Angles (θ_r_), Lattice Constants (*c*), Binding Energies (*E*_b_), Interlayer Distances (*d*), and Band Gaps (Gap) for Different AB-Stacking or Twisted Bilayer InSe/InSe

system	θ_r_ (degree)	*c* (Å)	*E*_b_ (meV)	*d* (Å)	gap (eV)
AB	0	4.09	32.86	2.90	0.86
√7	21.8	10.82	24.23	3.31	0.92
√13	32.2	14.74	23.37	3.39	0.93
√19	13.2	17.82	23.04	3.27	0.89

The band structures and projected density of states (PDOS) for AB-stacking and twisted InSe/InSes are shown in Figure 1e–h. It is found that all the twisted InSe/InSes are indirect band gap semiconductors with their band gaps around ∼0.90 eV, which is in accord with the previous results of bilayer InSe^51^ and almost insensitive to the twist angles. Such an electronic feather makes the InSe/InSe bilayer a stable candidate for data storing, optical catalysts, etc. Figure 2 plots the electrostatic potential of all the bilayer InSe/InSe systems. Similar to the AB-stacking MoS_2_ bilayer,^52^ a spontaneous polarization between the two InSe monolayers is found for AB-stacking bilayer InSe/InSe (see Figure 2a), and there is a potential drop of 0.11 eV along the *z* direction between the top and bottom InSe monolayer, indicating that building an electric field in the AB-stacking configuration will break the symmetry of InSe bilayer conformation and split the electronic states from two monolayers, as shown in the PDOS in Figure 1. As for the twisted systems with θ_r_ = 21.8, 32.2, and 13.2°, the energy states of the top InSe layer are the perfect superposition of those in the bottom layer due to their large interlayer distance, indicating that the intrinsic polarization at the interface and the interlayer interaction are much smaller than those of the AB-stacking one (see Figure 1f–h). Accordingly, the electrostatic potential of the two separate InSe layers is nearly the same with a potential drop of less than 0.01 eV (Figure 2b–d).

### 3.2 Gr/InSe Heterobilayer

**Figure 3:**
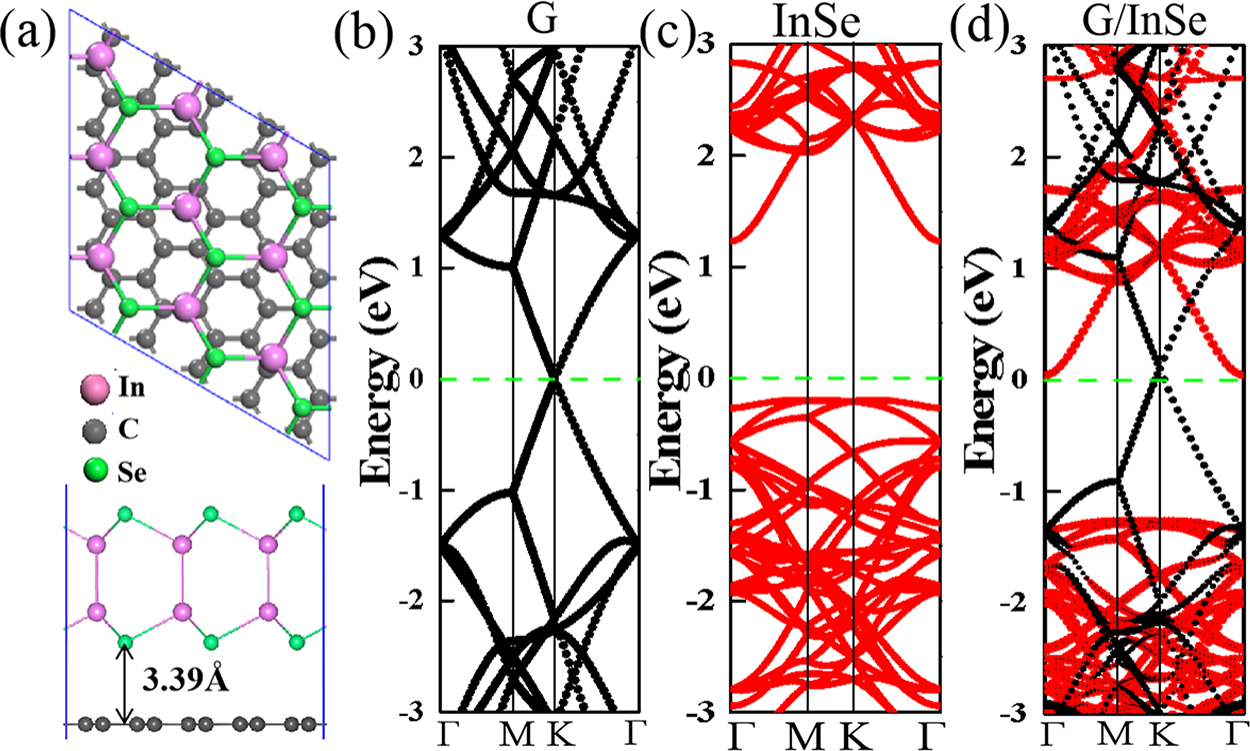
(a) Optimized structures and charge density difference of non-twisted Gr/InSe. (b–d) Band structures of the isolated 5 × 5 graphene supercell, 3 × 3 InSe supercell, and Gr/InSe supercell, respectively.

For the heterobilayer Gr/InSe, the non-twisted structure and three twisted ones with θ_r_ = 42.5, 52.4, and 13.9° are considered. For the non-twisted structure, the rhombus lattice parameters along the *a*/*b* vector direction are 12.30 Å, which is composed of the 5 × 5 graphene supercell and 3 × 3 InSe supercell with lattice mismatches for the graphene and InSe layer of only 0.28 and 0.29%, respectively (see Figure 3a).

Similarly, to determine the influence of the stacking order between the graphene and InSe layer, three types of configurations are considered: (i) Gr/InSe with one Se atom sitting on top of one C atom in graphene (Se–C), (ii) Gr/InSe having one In atom locating atop of one C atom in graphene (In–C), and (iii) Gr/InSe with one Se atom above the hollow site of graphene (Se–H) (see Figure S5 in the Supporting Information). It is found that almost no differences are found for the structures and electronic properties of these three configurations. First, the energy differences of these three isomers are very small (<3.3 meV per unit cell) and the interlayer distances of them are around 3.39 Å. Compared to freestanding graphene and InSe monolayers, the electronic structure of Gr/InSe can be regarded as a superposition of the energy states from each constituent (Figure 3b–d); that is, the graphene “layer” is still semimetal and the InSe “layer” is still a semiconductor with an indirect band gap of 1.31 eV. Similarly, the electronic properties of such a Gr/InSe heterostructure are also insensitive to the bilayer stacking orders (see Figure S5), and based on this, we only used the Se–C configuration to explore the structural and electronic properties of these Gr/InSe heterostructures.

To evaluate the coupling interaction between graphene and InSe, we calculated the *E*_b_ between different configurations based on the following formula
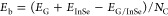
3where *E*_G/InSe_, *E*_G_, *E*_InSe_, and *N*_C_ are the energies of Gr/InSe, monolayer graphene, InSe, and the number of C atoms per unit cell, respectively. The calculated *E*_b_ for non-twisted Gr/InSe is 36.5 meV per C atom (as listed in Table 2), which is in the same order of magnitude as that in other vdW graphene-based heterostructures,^53,54^ indicating the weak van der Waals interaction between the graphene and InSe monolayer.

**Table 2:** Interlayer Rotation Angles (θ_r_), Lattice Constants (*c*), Binding Energies (*E*_b_), Lattice Strains (ε), and Interlayer Distances (*d*) for Different Gr/InSe Heterostructures

system (*p*:*q*)	θ_r_ (degree)	*c* (Å)	*E*_b_ (meV)	ε_InSe_ (%)	ε_G_ (%)	*d* (Å)
5:3	0	12.30	36.5	0.29	–0.28	3.36
6:√13	13.9	14.77	33.0	0.21	–0.21	3.38
√19:√7	42.5	10.79	36.2	–0.28	0.28	3.35
√43:4	52.4	16.27	38.9	–0.53	0.54	3.32

**Figure 4:**
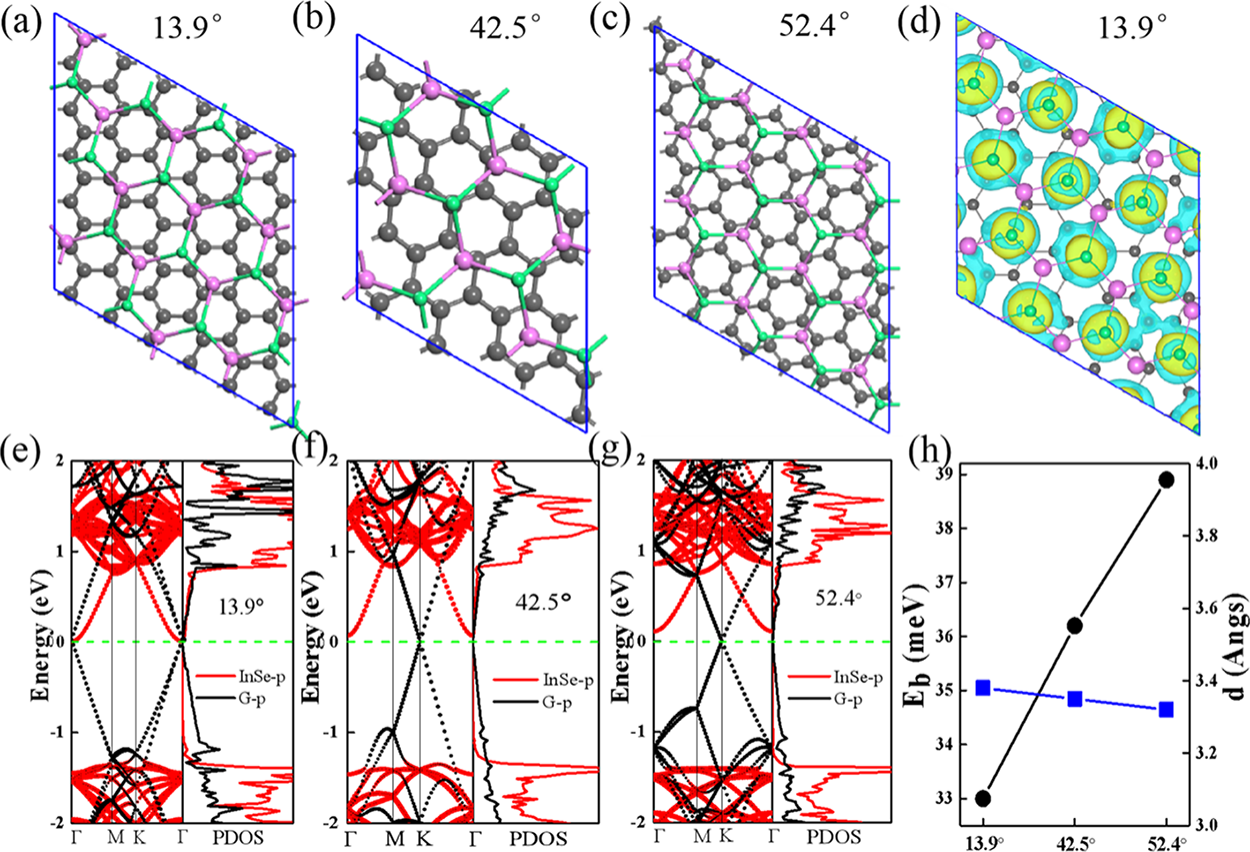
(a–c) Optimized structures, band structures, and PDOS (e–g) of twisted Gr/InSes heterostructures with θ_r_ = 13.9, 42.5, and 52.4°, respectively. (d) CDD plots of the Gr/InSe interface with a *p*:*q* value of 6:√13 (θ_r_ = 13.9°); yellow and blue colors represent the accumulation and depletion of electrons, respectively. The isosurface value is 1.0 × 10^–4^ e/bohr.^3^ (h) Binding energy (*E*_b_) and interlayer distance (*d*) for various twisted Gr/InSe bilayers. Black and blue lines represent *E*_b_ and *d*, respectively.

The top view of the three optimized twisted Gr/InSe bilayers with twist angles of 42.5, 52.4, and 13.9° are shown in Figure 4a−c, which have *p*:*q* values of 6:√13, √19:√7, and √43:4, respectively. The interlayer rotation angles, lattice mismatches, binding energies, and distances between graphene and InSe are listed in Table 2. Nearly perfect lattice matches are identified for these twisted Gr/InSe bilayers, in which the largest lattice mismatch is only 0.54% for the √43:4 supercell and the smallest lattice mismatch of 0.21% is found for the 6:√13 supercell. Moreover, the binding energies and interlayer distances of such twisted Gr/InSes are close to those of non-twisted ones (Figure 4h), namely, *E*_b_ ranges from 33.0 to 38.9 meV per C atom and *d* is in the range of 3.32–3.38 Å, which is in comparison with the interlayer distances of other InSe van der Waals heterostructures^25,55,56^ and independent on the interlayer rotation angles.

**Figure 5:**
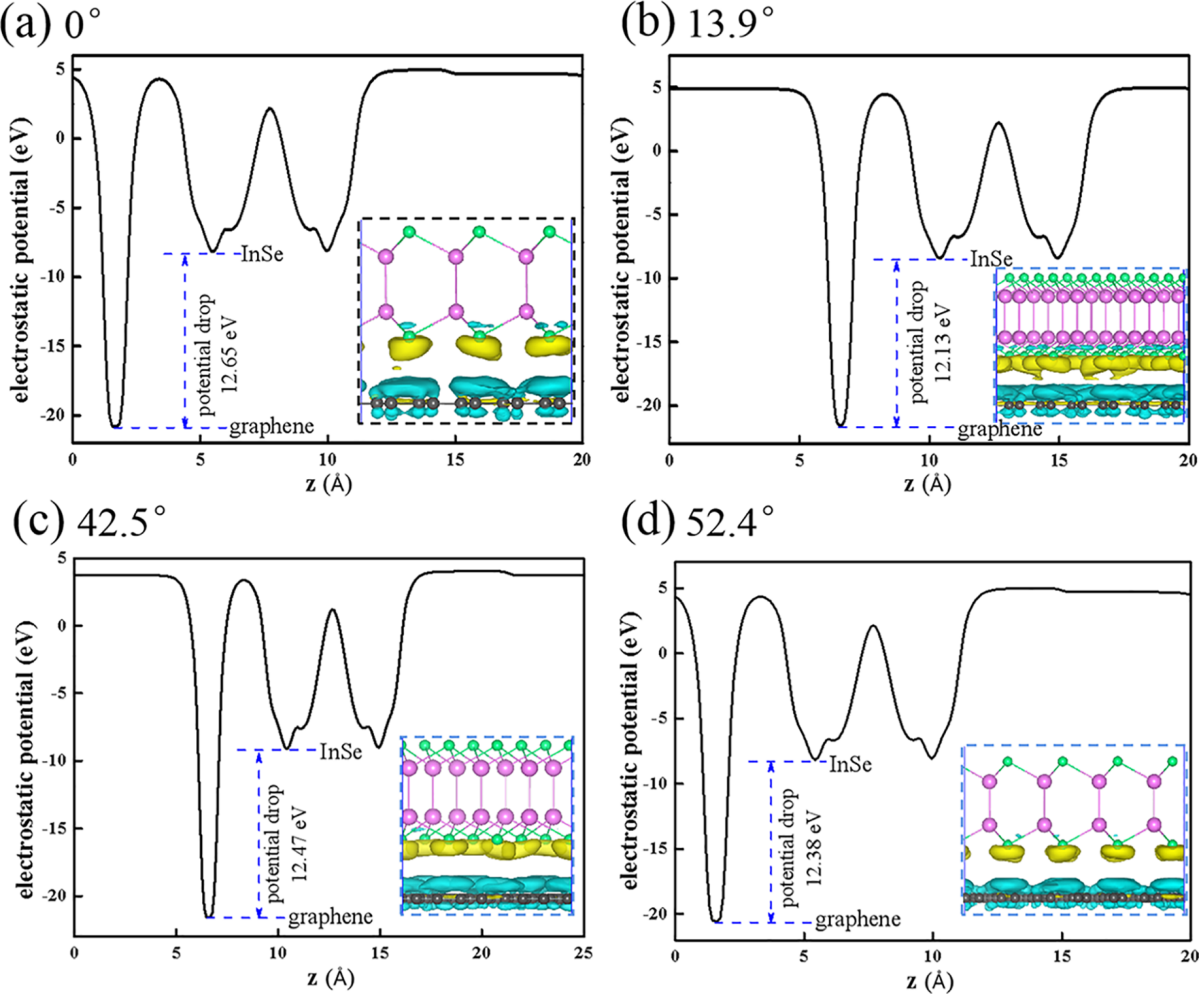
Electrostatic potential of the Gr/InSe bilayer along the *z* direction with different interlayer rotation angles of (a) 0°, (b) 13.9°, (c) 42.5°, and (d) 52.4°. Insets are CDDs for twisted Gr/InSe bilayers with different rotation angles; yellow and blue regions denote electron accumulation and depletion, respectively. The isosurface value is 1.0 × 10^–4^ e/bohr.^3^

One reason for the electronic property variation of the rotated bilayer Gr/InSe is that the interlayer coupling varies with different rotation angles as the states coupled in the two layers occur at different momentum.^12^ Comparisons of the CDDs at the interface of non-twisted and twisted Gr/InSes are shown in Figures 4d and 5, respectively. Bader charge analysis suggests that the depletion of electrons on graphene is about 7.7 × 10^–4^ e per C atom for the non-twisted one, which is decreased to be 6.4 × 10^–4^ e, 5.7 × 10^–4^ e, and 5.7 × 10^–4^ e per C atom for twisted Gr/InSe with misorientation angles of 13.9, 42.5, and 52.4°, respectively. The other reason is the electric potential difference between layers when rotation occurs. This can be verified by the electrostatic potential of the Gr/InSe heterostructure along the *z* direction (Figure 5), and a large potential drop of about 12.13–12.65 eV between the graphene and InSe layer in the heterostructures leads to the charge transfer from graphene to InSe due to the built-in electric field. The band structures and PDOS plots of twisted Gr/InSe heterobilayers are shown in Figure 4e–g. On one hand, similar to non-twisted Gr/InSe, the band structures of the graphene layer and InSe layer are well preserved in these hybridized structures. On the other hand, the indirect band gap of the “InSe” layer is found to vary with the twist angle, which are around 1.33, 1.47, and 1.49 eV at θ_r_ = 13.9, 42.5, and 52.4°, respectively, indicating that twisting is an effective way to tune the electronic properties of Gr/InSe.

## 4 Conclusions

In summary, we systematically studied the structures and electronic properties of twisted bilayer InSe/InSe and heterobilayer Gr/InSe by using first-principles calculations. For bilayer InSe/InSe, the band structures of AB-stacking InSe/InSe are different from their twisted isomers due to the spontaneous polarization between two InSe layers, while for the twisted systems, almost identical band gaps are found. As for the twisted Gr/InSe heterostructure, its band gaps are found to vary with the interlayer misorientation angles. Our results propose an effective way to tune the electronic properties of InSe, supplying useful information for designing novel electronic or optical devices based on InSe van der Waals structures.

## Supporting Information Available

Schematic plot of the skewed supercell in the hexagonal lattice with different rotation angles, the structure of AA-stacking bilayer InSe/InSe, charge density differences of the twisted bilayer InSe/InSe, and structures and band structures of the bilayer InSe/InSe and G/InSe with different interlayer translations (PDF)
