# Supplementary material for: Electronic Structures of Twisted Bilayer InSe/InSe and Heterobilayer Graphene/InSe
Source: ACS Omega. 2021 May 11;6(20):13426–32. doi: 10.1021/acsomega.1c01562 (PMC8158824; doi:10.1021/acsomega.1c01562)
Supplement: Supplementary file 1 — ao1c01562_si_001.pdf [file ao1c01562_si_001.pdf]

Supporting Information

Electronic Structures of Twisted Bilayer InSe/InSe and Hetero-Bilayer Graphene/InSe

Xiaojing Yao,[1] Xiuyun Zhang[2*]

[1]Department of Physics, Hebei Normal University, Shijiazhuang 050024, China

[2]College of Physics Science and Technology, Yangzhou University, Yangzhou 225002, China

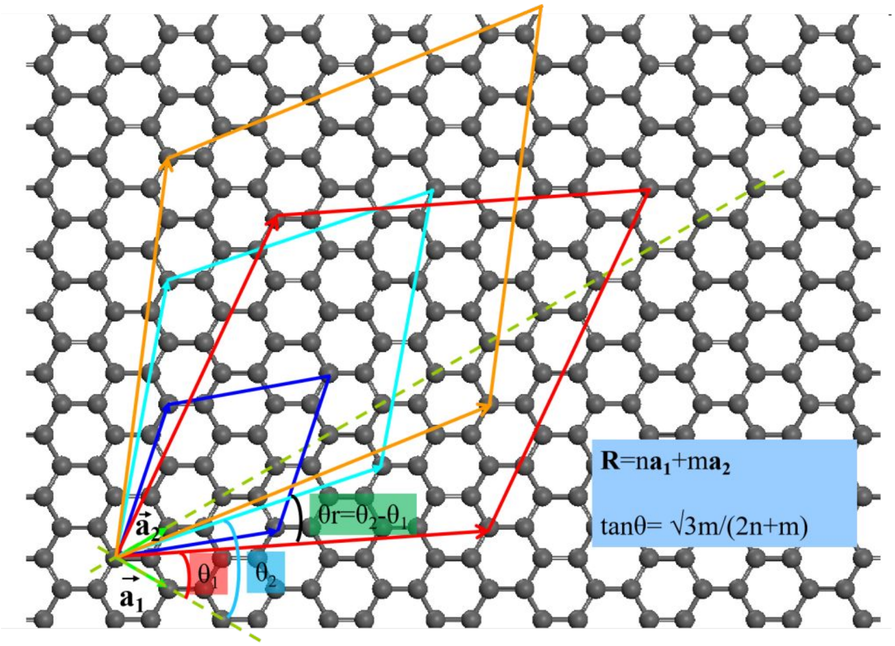

Figure S1. Schematic plot of the skewed supercell in hexagonal lattice with different rotation angles. The lattice vector is (n**a₁**+m**a₂**), and the skewed angle can be expressed as $\theta = \tan^{-1}\left(\frac{\sqrt{3}m}{2n+m}\right)$, the formed twisted bilayer has the rotation angle of $\theta r = \theta_2 - \theta_1$.

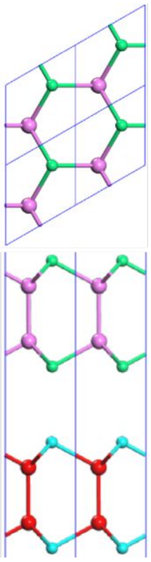

Figure S2. The structure of AA-stacking bilayer InSe/InSe.

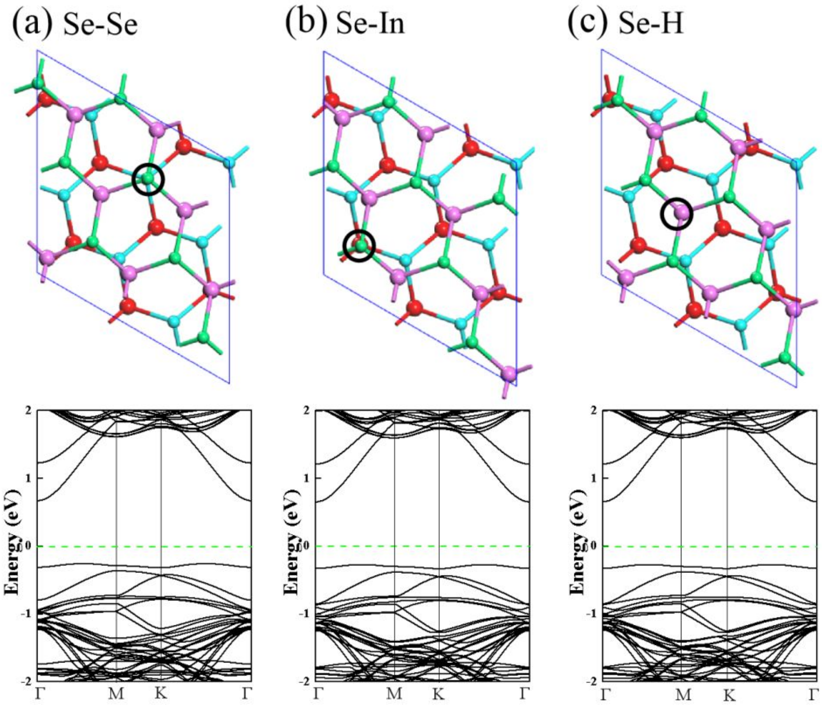

Figure S3. Structures and band structures of the twisted bilayer InSe/InSe (θr=21.8°) with different interlayer translation: (a) Se-Se; (b) Se-In; (c) Se-H.

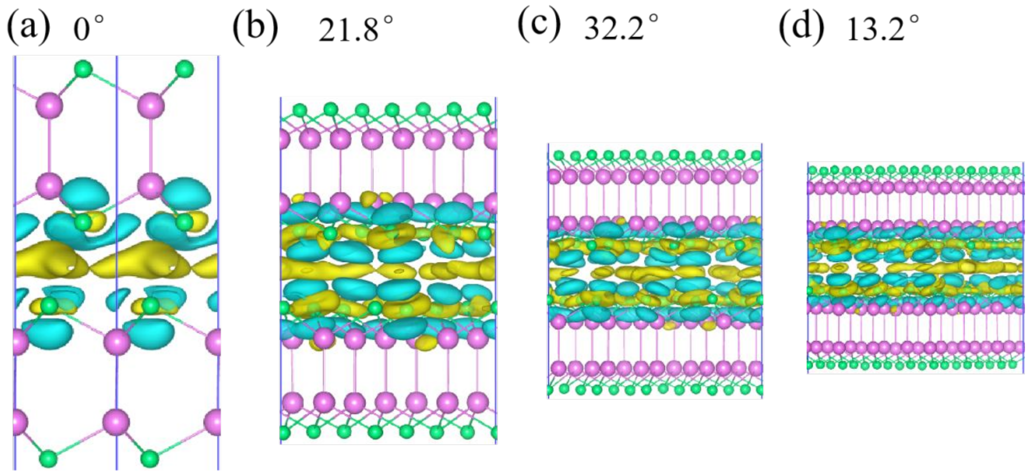

Figure S4. Side views of CDDs for twisted bilayer InSe/InSe with twist angles of (a) 0°, (b) 21.8°, (c) 32.2° and (d) 13.2°, respectively. Yellow and blue regions denote electron accumulation and depletion, respectively. The isosurface value is 1.2×10^{-4} e/bohr^3.

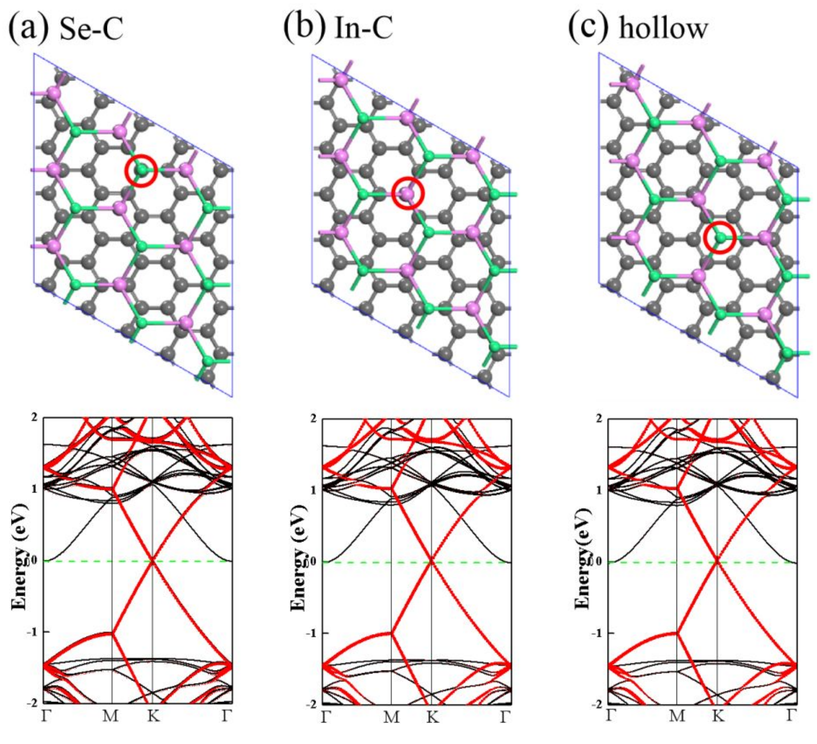

Figure S5. Structures and band structures of the bilayer G/InSe (θr=0°) with different interlayer translation: (a) Se-C; (b) In-C; (C) hollow.
